# Fluorescence quenching by high-power LEDs for highly sensitive fluorescence *in situ* hybridization

**DOI:** 10.3389/fnmol.2022.976349

**Published:** 2022-09-02

**Authors:** Yousuke Tsuneoka, Yusuke Atsumi, Aki Makanae, Mitsuru Yashiro, Hiromasa Funato

**Affiliations:** ^1^Department of Anatomy, Faculty of Medicine, Toho University, Tokyo, Japan; ^2^Center for Research and Product Development, Nepa Gene Co., Ltd., Chiba, Japan; ^3^International Institutes for Integrative Sleep Medicine (WPI-IIIS), University of Tsukuba, Ibaraki, Japan

**Keywords:** autofluorescence, *in situ* hybridization, hybridization chain reaction, lipofuscin, multiround ISH

## Abstract

Recent technical advances have made fluorescent *in situ* hybridization (ISH) a pivotal method to analyze neural tissue. In a highly sensitive ISH, it is important to reduce tissue autofluorescence. We developed a photobleaching device using a light-emitting diode (LED) illuminator to quench autofluorescence in neural tissue. This device was equipped with 12 high-power LEDs (30 W per single LED) and an evaporative cooling system, and these features achieved highly efficient bleaching of autofluorescence and minimized tissue damage. Even after 60 min of photobleaching with evaporative cooling, the temperature gain of the tissue slide was suppressed almost completely. The autofluorescence of lipofuscin-like granules completely disappeared after 60 min of photobleaching, as did other background autofluorescence observed in the mouse cortex and hippocampus. In combination with the recently developed fluorescent ISH method using the hybridization chain reaction (HCR), high signal/noise ratio imaging was achieved without reduction of ISH sensitivity to visualize rare mRNA at single copy resolution by quenching autofluorescence. Photobleaching by the LED illuminator was also effective in quenching the fluorescent staining of ISH-HCR. We performed multiround ISH by repeating the cycle of HCR staining, confocal imaging, and photobleaching. In addition to the two-round ISH, fluorescent immunohistochemistry or fluorescent Nissl staining was conducted on the same tissue. This LED illuminator provides a quick and simple way to reduce autofluorescence and quench fluorescent dyes for multiround ISH with minimum tissue degradation.

## Introduction

Detecting multiple molecules using fluorescent labeling is crucial to many fields in biology. In this decade, various techniques were developed to detect mRNAs at single-molecule resolution, and they were characterized by two aspects: signal intensity and signal specificity. Nonenzymatic signal amplification was achieved by sequential hybridization resulting in large branched-DNA (Player et al., 2001; Wang et al., 2012; RNAscope^®^ or viewRNA^TM^) or hybridization chain reaction (HCR) of hairpin DNAs (Dirks and Pierce, 2004; Choi et al., 2010). Polymerase-based signal amplification is known as rolling cycle amplification (Larsson et al., 2010). Although there are various methods for achieving high signal intensity, the common strategy to ensure signal reliability is a specific reaction that relies on the matching of two split sequences to a single target sequence (Larsson et al., 2010; Wang et al., 2012; Choi et al., 2018). This approach can drastically suppress the noise derived from the nonspecific binding of probes. Another considerable noise for imaging fluorescently stained tissue is autofluorescence, which cannot be ignored even if the above-listed highly-sensitive ISH methods were used (Rocco et al., 2017; Baharlou et al., 2021; Maynard et al., 2021).

Autofluorescence is derived from lipofuscin, hemosiderin, collagen and elastin fibers, and other metabolic products of cells (O’Connell et al., 1986; Schnell et al., 1999; Billinton and Knight, 2001). Although the causes of autofluorescence seem to be diverse, the major source of autofluorescence in nervous tissue has been known to be lysosome-associated granule accumulation, lipofuscin (Ivy et al., 1984). Substances emitting autofluorescence, including lipofuscin, have a broader range of fluorescent emission spectra than those of commonly used fluorescent dyes. Although autofluorescence signals can be separated from fluorescent labeling by spectrum analysis (Pyon et al., 2019), this is not practical in cases where autofluorescence is stronger than the target signal or in multiple staining using different fluorescent dyes. Various reagents that quench autofluorescence have been reported, such as CuSO_4_ in ammonium acetate buffer, Sudan Black B, NaBH_4_, and other commercially available quenchers (Clancy and Cauller, 1998; Schnell et al., 1999; Rocco et al., 2017). However, these reagents can quench genuine fluorescence derived from dyes or induce backgrounds in a specific fluorescent spectrum (Schnell et al., 1999; Sun and Chakrabartty, 2016; Rocco et al., 2017; unpublished observation).

Another approach to eliminate autofluorescence from tissue is light irradiation to induce irreversible modification of fluorophores. Irradiation by multiple fluorescent tubes consisting of UV and visible light effectively reduced autofluorescence, but it required cooling of specimens and long irradiation times (24 h or longer; Neumann and Gabel, 2002). Irradiation by a mercury arc lamp with an emission spectrum from long UV to be visible through an objective lens equipped with a fluorescence microscope successfully quenched autofluorescence within only 20 min, although the quenched area was limited within the field of view of the microscope (Neumann and Gabel, 2002). Others also tried to reduce autofluorescence using a light-emitting diode (LED). Illumination by LED arrays also eliminates tissue autofluorescence, but it takes 1–3 days (Duong and Han, 2013; Sun and Chakrabartty, 2016; Ku et al., 2020). When the LED array got closer to the specimens, the effectiveness of quenching autofluorescence became greater, but the specimens became too warm (Duong and Han, 2013). Photobleaching has not become a major method to reduce autofluorescence, perhaps because of these problems of heat generation and prolonged irradiation.

To solve the problems in photobleaching autofluorescence in tissues, we have developed a highly efficient photobleaching device using LED illumination. The LED illuminator is equipped with high-power LEDs and an evaporative cooling system to reduce autofluorescence while preventing tissue damage induced by heat. This device is especially useful for *in situ* hybridization (ISH) using HCR amplification because it dramatically improves the signal/noise ratio in histological examination. HCR is an enzyme-free polymerization of two kinds of hairpin DNAs in the presence of an initiator nucleotide (Dirks and Pierce, 2004). In ISH-HCR, fluorescently labeled hairpin DNAs are amplified by initiator nucleotides with probe sequences that hybridize target mRNAs (Choi et al., 2010, 2014, 2018). Recently, we improved the ISH-HCR method by using short hairpin DNAs (Tsuneoka and Funato, 2020). ISH-HCR using short hairpin DNAs does not require high temperature or proteinase treatment, thus causing minimum tissue damage and allows the detection of endogenous fluorescently tagged proteins (Maejima et al., 2021). Furthermore, the LED illuminator effectively bleached fluorescent labeling by ISH-HCR. Using this quenching method, we also established simple multiround fluorescent ISH combined with immunohistochemistry (IHC) by repeating cycles of fluorescent staining, confocal imaging, and quenching. Using this method, we succeeded in visualizing nine molecules in the same brain section.

## Materials and Methods

### Animals

All animal procedures were conducted in accordance with the Guidelines for Animal Experiments of Toho University and were approved by the Institutional Animal Care and Use Committee of Toho University (Approval Protocol ID #21-53-405). Breeding pairs of C57BL/6J mice were obtained from Japan SLC and CLEA Japan. Mice were raised in our breeding colony under controlled conditions (12 h light/dark cycle, lights on at 8:00 A.M., 23 ± 2°C, 55 ± 5% humidity, and *ad libitum* access to water and food).

Twelve- to 15-week-old male mice were anesthetized with sodium pentobarbital (50 mg/kg, i.p.) and then transcardially perfused with 4% paraformaldehyde (PFA) in phosphate-buffered saline (PBS). The brains were postfixed in 4% PFA at 4°C overnight, followed by cryoprotection in 30% sucrose in PBS for 2 days, embedded in Surgipath (FSC22, Leica Biosystems), and stored at −80°C. The frozen brains were cryosectioned coronally at a thickness of 10, 20, or 40 μm. The sections were mounted on adhesive-coated slide glass (Platinum Pro, Matsunami, Japan) and stored at −20°C until use. Ten-μm-thick sections were used for multiround ISH/IHC (“Multiround ISH-HCR and IHC” Section) and 20–40-μm-thick sections were used for quenching autofluorescence (“Quenching autofluorescence” and “Single round ISH-HCR” Section).

### Equipment

**Figure 1:**
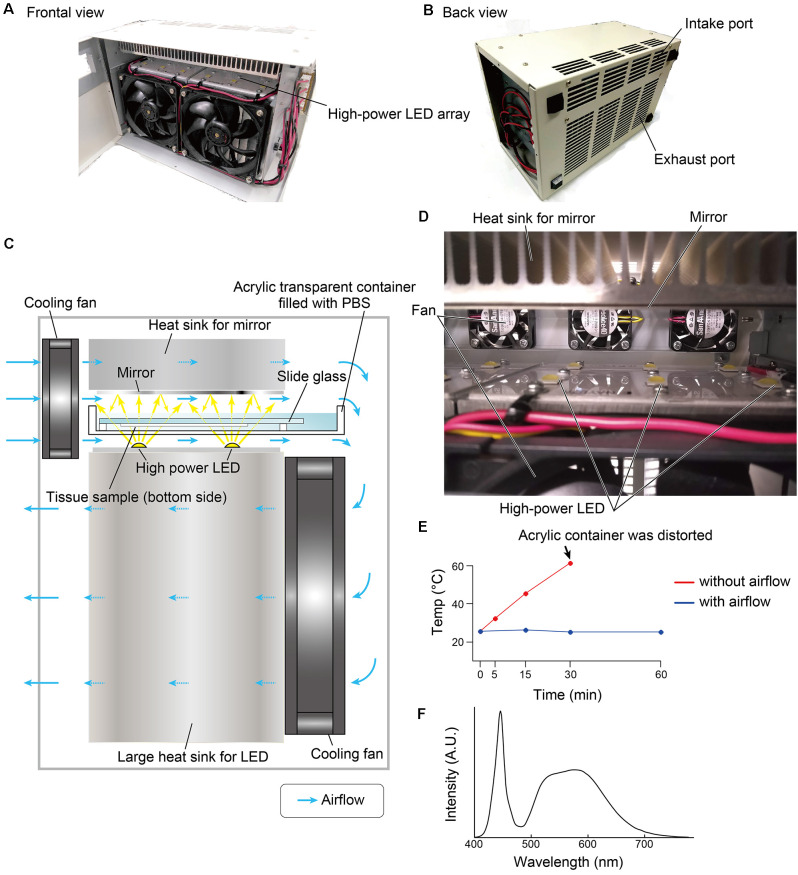
Overview of the LED illuminator, TiYO^TM^. **(A)** Frontal view and **(B)** back view of the LED illuminator. **(C)** Lateral illustration of the LED illuminator. Two high-power LEDs (more than 3,000 lumens/single LED) are placed in one column, and the LEDs are arrayed as 2 × 6. LED circuit boards are mounted on a large heat sink (12 × 24 × 12 cm). On the upper side of the sample, a mirror with a heat sink (3 × 24 × 12 cm) is placed. Two heat sinks are cooled by cooling fans. The airflow shown by the blue arrow also removes heat from the buffer solution in the acrylic container. **(D)** Inside view of the LED illuminator. **(E)** Temperature change of the buffer solution inside the acrylic container according to the duration of photoirradiation. **(F)** Spectrum of the LED used in this study.

The overall design of the LED illuminator is described in Figure 1. The LED illuminator TiYO^TM^, meaning the sun in Japanese, was designed to illuminate each slide glass with two high-power LEDs. The LEDs were placed as a 2 × 6 array, and the distance interval was 4 cm. In our preliminary trial, the most efficient quenching in the brain tissue was observed in the blue LED (dominant spectrum: 475 nm), so we chose a cool-white LED (NV9W149AM, Nichia Corporation, Japan): the nominal correlated color temperature is 5,000 K, and the luminous flux is 4,131 lm at a 3,000-mA drive current. A mirror (polished aluminum plate) was placed 24 mm from the LED to illuminate the slide glass evenly. The minimum distance between the LED and the tissue section was set at 5 mm, and the slides were placed in a transparent acrylate container (15 × 260 × 105 mm) filled with PBS. The LED board and the mirror were equipped with heat sinks (120 × 240 × 120 mm for the LED board and 30 × 240 × 120 for the mirror). The LED illuminator was also equipped with four small cooling fans (airflow: 5.3 cfm, SanAce40-109P0412H901, SANYO, Japan) and two large cooling fans (airflow: 70.6 cfm, SanAce120-9S1212F401, SANYO, Japan) to minimize changes in the tissue temperature (Figure 1E). The temperature change was measured at three positions close to the slide glass by thermocouple.

### Quenching autofluorescence

To examine the efficiency of the LED illuminator described in Section “Equipment”, the brain sections without any staining procedures were bleached for 0, 15, 30, 60, or 90 min in an acrylate container filled with PBS. The LEDs were driven at a constant current as the maximum power output (30 W for a single LED). After bleaching, the slides were coverslipped with VECTASHIELD Vibrance (Vector Laboratories, H-1700) and observed by confocal microscopy (“Histological analysis”).

### Single round ISH-HCR

#### Preparation of probes and fluorescent hairpin DNAs

The probes for ISH-HCR were designed to minimize off-target complementarity using a homology search by NCBI Blastn^1^, and they were designed to have split-initiator sequences with an mRNA binding site. The number of target sites per single mRNA varied from 5 to 30 (Supplementary Table 1) based on their abundance. The DNA probes were synthesized as standard desalted oligos (Integrated DNA Technologies). The fluorescent hairpin DNAs were prepared by conjugation of succinimidyl ester of fluorophores (Sarafluore488, ATTO550, or Cy5) and synthesized with a C12 amino-linker at the 5’ end (Integrated DNA Technologies or Tsukuba Oligo Services). The DNA probes and hairpin DNAs were purified by denaturing polyacrylamide gel electrophoresis using 20% polyacrylamide gels. The detailed procedures for DNA purification were described in our previous study (Tsuneoka and Funato, 2020).

#### Probe hybridization

ISH procedures were similar to those previously described with some modifications (Tsuneoka and Funato, 2020; Katayama et al., 2022). The sections were washed with PBS containing 0.2% Triton (PBST) and immersed in methanol for 10 min, followed by PBST washing for 5 min twice. After washing, the sections were prehybridized for 5 min at 37°C in a hybridization buffer containing 10% dextran sulfate, 0.5× SSC, 0.1% Tween 20, 50 μg/ml heparin, and 1× Denhardt’s solution. The sections were treated with another hybridization solution containing a mixture of 10 nM probes for *Drd1*, *Drd2*, and *Vglut1* mRNAs, and these probes contained the split-initiator sequences for amplification of S41, S23, and S10 hairpin DNAs (Supplementary Table 1), respectively. The slides with hybridization solution were covered by a plastic film (Labo-pita, NIPRO, Japan) to prevent evaporation and incubated overnight at 37°C. After hybridization, the sections were washed three times for 10 min in 0.5× SSC containing 0.1% Tween 20 at 37°C. Then, the sections were bleached by an LED illuminator for 60 min in PBST. For comparison, the other sections from the same animals were immersed in PBST without bleaching. The control experiment was performed without probes.

#### HCR amplification

For HCR amplification, SaraFluor488-conjugated S10, ATTO550-conjugated S41, and Cy5-conjugated S23 hairpin DNA pairs were used (Supplementary Table 1). These hairpin DNA solutions were separately snap-cooled (heated to 95°C for 1 min and then gradually cooled to 65°C for 15 min and 25°C for 40 min) before use. The sections were incubated in an amplification buffer (10% dextran sulfate in 8× SSC, 0.2% Triton X-100, 100 mM MgCl_2_) for 5 min and then immersed in another amplification buffer containing 60 nM hairpin DNA for 2 h at 25°C. The samples were washed with PBST, followed by staining with Hoechst 33342 (2 μg/ml, Dojindo, H342) for 15 min at room temperature. After washing with PBS, the slides were coverslipped with VECTASHIELD vibrance.

### Multiround ISH-HCR and IHC

#### Probe hybridization

For multiround ISH, the procedures for probe hybridization were the same as those for single-round ISH (“Preparation of probes and fluorescent hairpin DNAs” Section) except for probe number, and the hybridization buffer contained six probes (probe set 1: Drd1-S41, Drd2-S23, Vgat-S68, Vglut2-S45, Penk-A161, and Pdyn-S85; probe set 2: Esr1-S86, Moxd1-S73, Hrh1-S10, Tac2-S81, Cart-S83, and Neurotensin-S72, Supplementary Table 1). A control experiment was performed without probes. After hybridization and subsequent washing, the sections were bleached by an LED illuminator for 60 min in PBST to quench autofluorescence.

#### HCR amplification and quenching fluorescent labeling

The first HCR reaction was performed using three of six hairpin DNA pairs. For probe set 1, SaraFluor488-conjugated S41, ATTO550-conjugated S23, and Cy5-conjugated S68 hairpin DNAs were used. For probe set 2, SaraFluor488-conjugated S86, ATTO550-conjugated S10, and ATTO647N-conjugated S73 hairpin DNAs were used (Supplementary Table 1). After 2 h of HCR amplification followed by Hoechst 33342 nuclear staining, the sections were coverslipped with 50% glycerol/PBS. Fluorescence images were obtained by confocal microscopy (“Histological analysis” Section), and then, the sections were bleached by the LED illuminator for 120 min in PBST. At this time, the cover glass was separated from the sections.

After a brief examination by fluorescence microscopy to confirm the extinction of the fluorescent signals, the sections were subjected to the second HCR reaction using the remaining three hairpin DNA pairs. For probe set 1, SaraFluor488-conjugated S45, ATTO550-conjugated A161, and Cy5-conjugated S85 hairpin DNAs were used. For probe set 2, SaraFluor488-conjugated S81, ATTO550-conjugated S83, and Cy5-conjugated S72 hairpin DNAs were used (Supplementary Table 1). After 2 h of HCR amplification without additional nuclear staining, the sections were coverslipped with 50% glycerol/PBS. The fluorescent images in a similar location to the first imaging were obtained by confocal microscopy (“Histological analysis”), and then, the sections were bleached by the LED illuminator for 120 min in PBST.

#### IHC and fluorescent Nissl staining

After a brief examination by fluorescence microscopy to confirm the extinction of the fluorescent signals, the sections stained by probe set 1 proceeded to IHC, and the sections stained by probe set 2 proceeded to fluorescent Nissl staining. For IHC, the sections were blocked with 0.8% BlockAce (Dainihon-Seiyaku, Japan) in PBST for 30 min. Then, a cocktail of mouse anti-tyrosine hydroxylase antibody, goat anti-oxytocin neurophysin antibody, and rabbit anti-nNos antibody in 0.4% BlockAce/PBST was applied to the sections and incubated at 4°C overnight in a humidified chamber. The sections were washed with PBST three times for 5 min and incubated in a cocktail of AlexaFluor488-conjugated anti-mouse IgG antibody, AlexaFluor568-conjugated anti-goat IgG antibody, AlexaFluor647-conjugated anti-rabbit IgG antibody and Hoechst 33342 for 60 min at room temperature. For fluorescent Nissl staining, the sections were treated with Neurotrace 640/660 (1:500, N21483, Thermo Fisher Scientific, USA) and Hoechst 33342 for 60 min. The sections were washed and coverslipped with 50% glycerol/PBS. The fluorescent images in a similar location to the first imaging were obtained similarly.

### Histological analysis

Fluorescent photomicrographs were obtained using a Nikon Eclipse Ni microscope equipped with the A1R confocal detection system under 20×/0.75 NA objective lenses at more than 8 μs/pixel speed at 0.314–1.28 μm/pixel resolution (Nikon Instruments Inc., Tokyo, Japan). The spectra passing through fluorescent filters were 425–475 (blue), 500–550 (green), 570–620 (red), and more than 650 nm (infrared). Tiled images were captured and automatically stitched by NIS-Elements C software (Nikon Instruments Inc., Tokyo, Japan). Z stacked images were captured with a distance of 1 μm between the optical slices.

Images were analyzed using ImageJ software (version 1.50i, NIH, USA) to adjust the contrast and to evaluate the overall fluorescence intensity, maximum intensity, and number of lipofuscin-like granules. Quantification of the fluorescent photographs was performed at the same threshold and adjustment of contrast. To evaluate quenching autofluorescence, four sets of sections, including the cingulate cortex, were used. Lipofuscin-like granules were counted on the thresholded image of the red filter. In the multiround ISH-IHC (Section “Multiround ISH-HCR and IHC” Section), the XY position of the images was adjusted manually based on the nuclear staining. To confirm quenching fluorescent dyes, the contours were set based on the threshold images captured before light irradiation by analyze particles menu command in the ImageJ. The signal to background ratio before and after photobleaching were compared by measuring mean intensity inside or outside the contours. In the colocalization analysis, cell boundaries were determined based on Nissl staining. If more than one punctum was observed within the single-cell boundary, the cells were judged as mRNA-positive cells.

## Results

### Quenching autofluorescence

**Figure 2:**
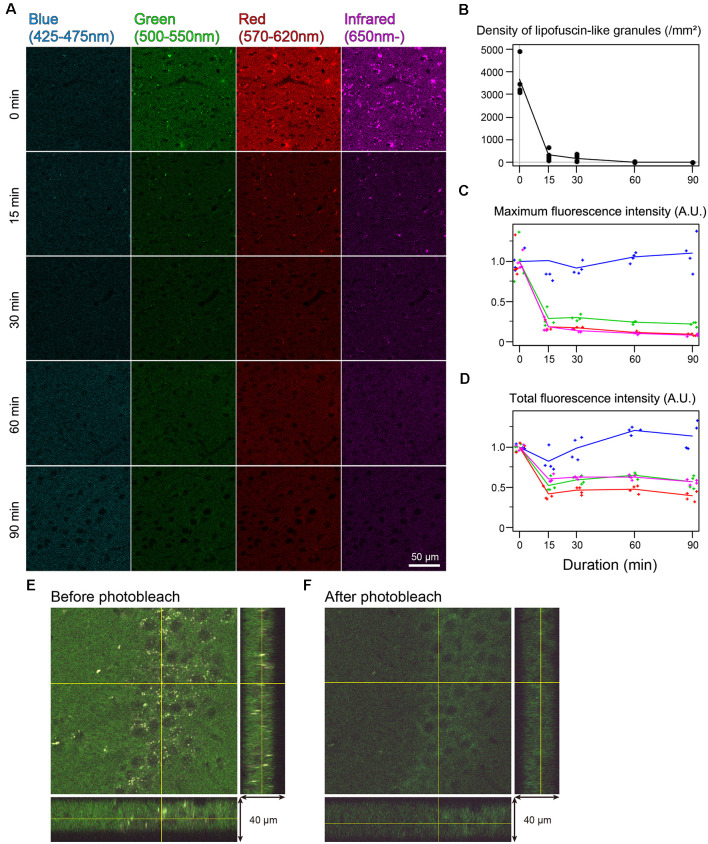
Rapid quenching of autofluorescence in the mouse cingulate cortex by photobleaching using TiYO^TM^. **(A)** Change in autofluorescence according to the duration of photobleaching. Each row shows photos from different specimens. Scale bar: 50 μm. **(B)** The density of lipofuscin-like granules. **(C)** Maximum fluorescence intensity. **(D)** Total fluorescence intensity. **(B–D)** Dots and lines represent the measured values and their means, respectively. Fluorescence intensities are shown as arbitrary units (A.U.). **(E,F)** Orthogonal views of the same 40 μm-thick section before **(E)** and after photobleaching **(F)**.

In the cingulate cortex as well as in other brain areas, autofluorescence of lipofuscin-like granules was abundantly observed around the cell nucleus of nonbleached sections. The fluorescent spectrum of these granules was distributed in green, red, and infrared but weak in blue (Figure 2A). The fluorescent signals of these granules decreased depending on the bleaching duration, and the signals completely disappeared after 60 min of bleaching (Figure 2A). The mean number of granules in the tissue bleached for 15 and 30 min changed to 8.33 ± 4.01 and 4.65 ± 2.22% from nonbleached tissue, respectively (Figure 2B). The maximum fluorescent intensities, except for the blue filter, dropped to 20%–30% after 15 min of bleaching and decreased slowly as time elapsed (Figure 2C). In addition to the lipofuscin-like structure, these bleaching effects were also observed across the tissue section. The total fluorescence intensity, including background fluorescence in the tissue, drastically decreased after 15 min of bleaching, except for the blue spectrum (Figure 2D). In the z-stack image of 40-μm-thick sections, there was no bias of photobleaching (Figures 2E,F).

### Single ISH-HCR with photobleaching

**Figure 3:**
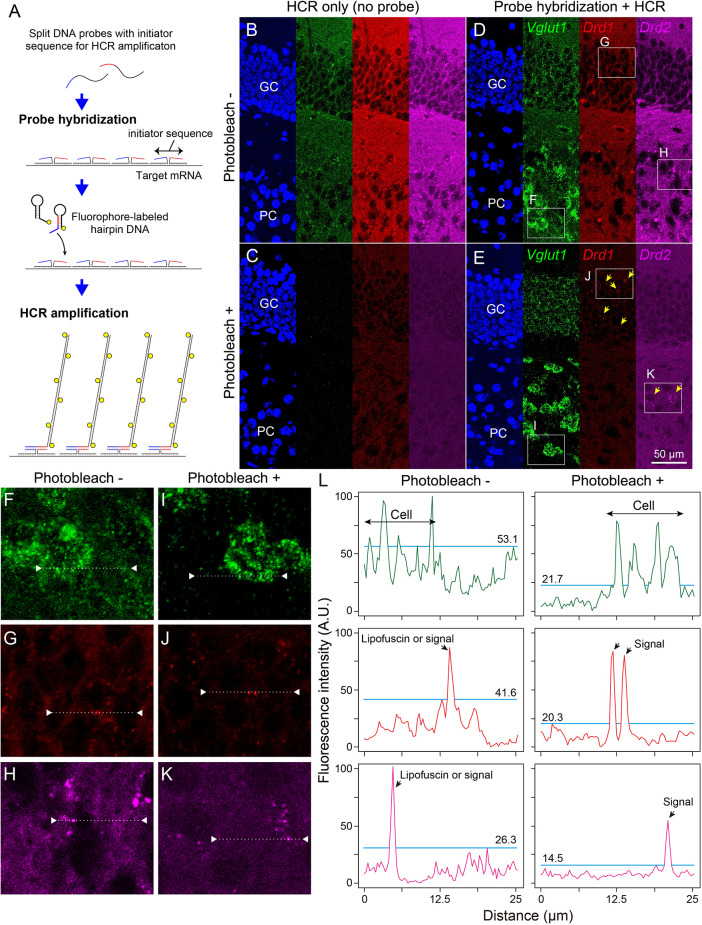
Effect of photobleaching on the signal/noise ratio of ISH-HCR staining. **(A)** Schematic drawings describing ISH-HCR staining in this study. ISH-HCR consists of two steps: hybridization of split DNA probes and HCR amplification of fluorophore-labeled hairpin DNAs. HCR amplification starts only in the case that both split probes are hybridized with target mRNA. In the HCR amplification step, a pair of fluorophore-labeled hairpin DNAs polymerize on the hybridized probes with complete initiator sequences. For details, see Tsuneoka and Funato (2020). **(B–E)** Representative photomicrograph of ISH-HCR staining for *Vglut1* (green), *Drd1* (red), and *Drd2* (magenta) mRNAs in addition to Hoechst 33342 nuclear staining (blue) in the dentate gyrus of the mouse hippocampus. No probe control without photobleaching **(B)** and with photobleaching **(C)**. ISH-HCR staining of *Vglut1*, *Drd1*, and *Drd2* without photobleaching **(D)** and with photobleaching **(E)**. Arrows indicate *Drd1*- or *Drd2*-positive cells. Scale bar: 50 μm. GC, granule cell layer; PC, polymorph cell layer. **(F–K)** Magnified views indicated by rectangles in **(D,E)**. **(L)** Line profile of fluorescent intensity indicated by dotted lines and triangles on **(F–K)**. Light blue lines indicate the maximum intensity of background signals. Fluorescence intensities are shown as arbitrary units (A.U.).

Next, we examined whether photobleaching before HCR staining improves the signal-to-noise ratio in the ISH-HCR of mouse hippocampal tissue. In the no-probe control, the fluorescent signals were observed as granules with a broad color spectrum, suggesting autofluorescence (Figure 3B). When the tissue slides were irradiated by the LED illuminator for 60 min, both granule-derived and background autofluorescence drastically decreased, and detectable signals were rarely found in the no-probe control section (Figure 3C). Although probe hybridization and subsequent HCR developed specific fluorescent labeling of *Vglut1* mRNA in the hippocampal sections, the staining results of *Drd1* and *Drd2* mRNA without photobleaching were not distinct from those of control sections (Figure 3D). When the sections were illuminated for 60 min, *Drd1*-positive cells could be moderately found as 1–3 puncta per cell in the granule cell layer, and a small number of *Drd2*-positive cells were also found in the pyramidal layer (Figure 3E). Line profile of fluorescent intensity showed that the background signals reduced to approximately half by photobleaching without a decrease in HCR signals (Figure 3L).

### Multiround ISH-IHC

**Figure 4:**
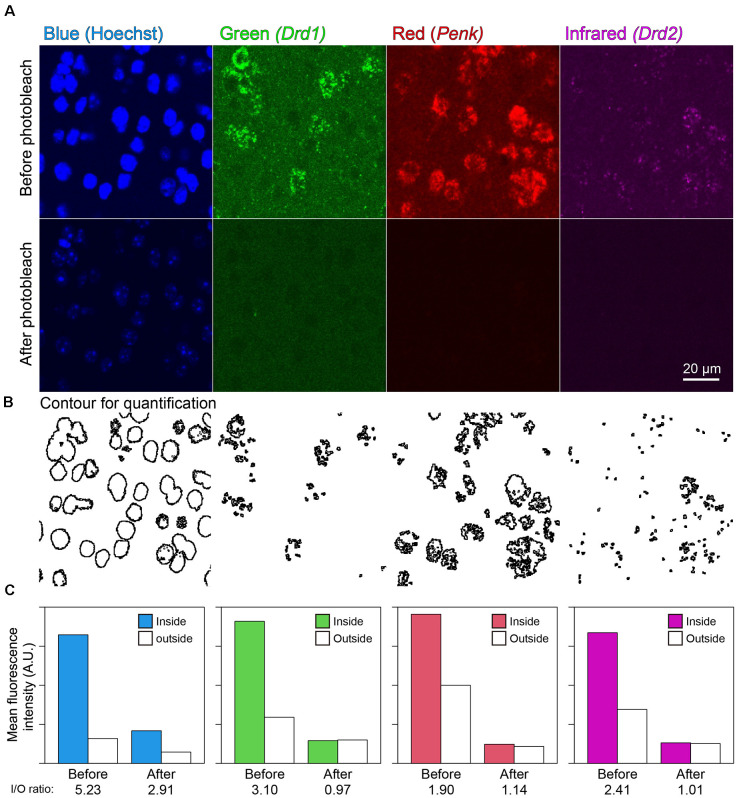
Effect of photobleaching on the fluorescence of ISH-HCR. **(A)** Mouse striatal tissue stained with *Drd1*, *Penk*, and *Drd2* mRNA in addition to Hoechst 33342 nuclear staining. The upper panels show confocal images before photobleaching, and the lower panels show confocal images in the same location after photobleaching. Scale bar: 20 μm. **(B)** Contour for quantification of signal to background ratio change shown in **(C)**. **(C)** Bar plots show mean fluorescence intensity inside or outside the contour. The inside/outside ratio of fluorescence intensity are shown at bottom line.

In the mouse striatum, ISH-HCR successfully detected *Drd1*, *Penk*, and *Drd2* mRNAs by SaraFluor488, ATTO550, and Cy5-labeled hairpin DNAs, respectively. Photobleaching by our LED illuminator for 2 h also diminished the fluorescence by these HCR stains to undetectable levels, while Hoechst 33342 nuclear staining did not completely disappear (Figures 4A,C). In addition, the signal to background ratio became close to 1.0, suggesting that the tissue fluorescence became uniform after photobleaching (Figures 4B,C).

**Figure 5:**
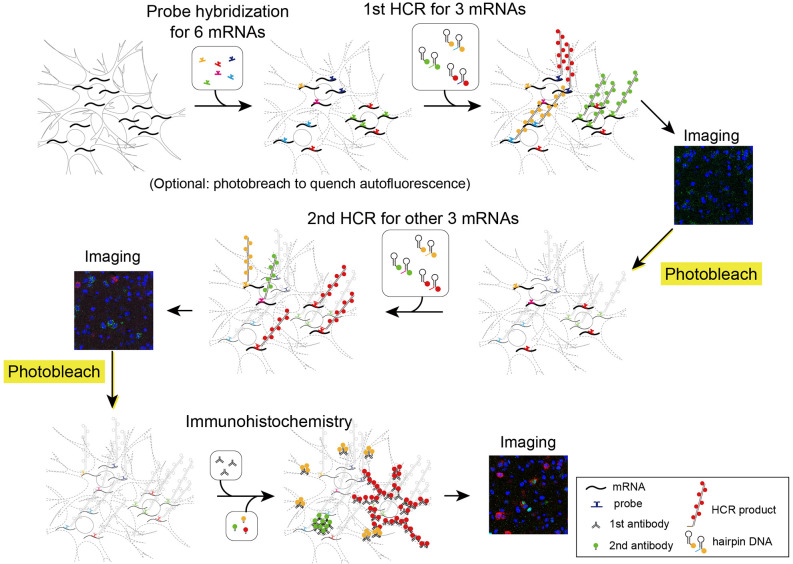
Schematic illustration describing two rounds of ISH combined with IHC. First, all probes of mRNAs of interest were hybridized simultaneously. The tissue was subjected to two rounds of staining by HCR for three mRNAs, confocal imaging, and photobleaching. Then, the tissue was subjected to IHC and confocal imaging.

**Figure 6:**
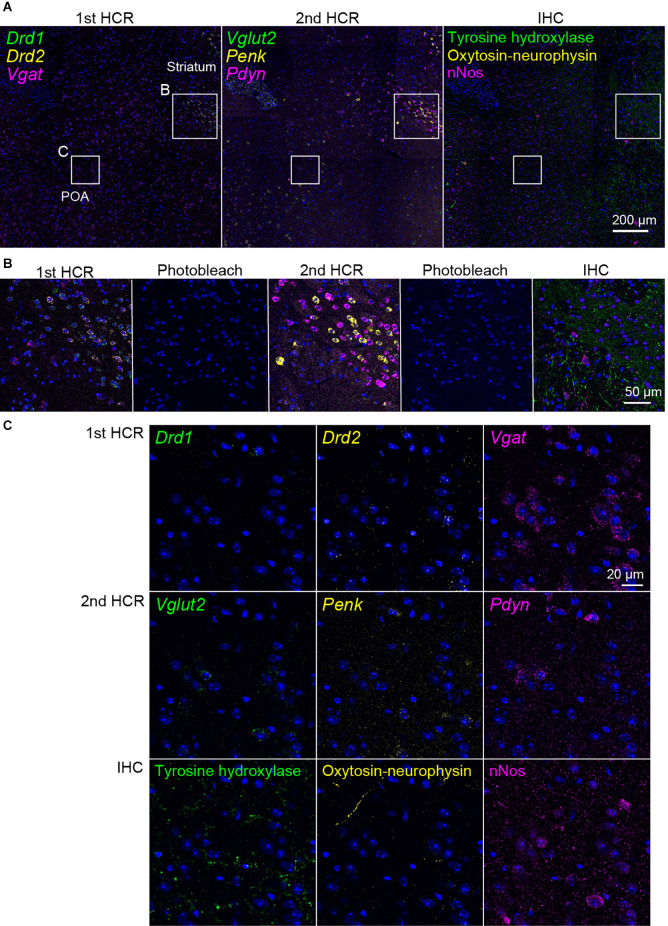
Representative photomicrographs of two-round ISH-IHC. The first HCR visualized *Drd1* (green, S41-SaraFluor488), *Drd2* (yellow, S23-Atto550), and *Vgat* (magenta, S68-Cy5) mRNAs. The second HCR visualized* Vglut2* (green, S45-SaraFluor488), *Penk* (yellow, A161-Atto550), and *Pdyn* (magenta, S85-Cy5) mRNAs. In the IHC, tyrosine hydroxylase, oxytocin-neurophysin, and nNos were visualized by Alexa488, Alexa568, and Alexa647 secondary antibodies, respectively. **(A)** Merged low-magnification images of mouse sections, including the preoptic area (POA) and striatum. Rectangles indicate the regions shown in **(B,C)**. Scale bar: 200 μm. **(B)** Merged, sequential images of the first HCR, photobleached image, the second HCR, photobleached image, and IHC. Scale bar: 50 μm. **(C)** Separate high-magnification images of each molecule. Scale bar: 20 μm.

Next, we performed two rounds of ISH-HCR combined with IHC (Figure 5). In the first HCR using S41-SaraFluor488, S23-ATTO550, and S68-Cy5 hairpin DNAs, the *Drd1*, *Drd2*, and *Vgat* mRNAs were observed intensely in the striatum, and relatively weak expression was observed in the preoptic area (Figure 6A). After photobleaching, no detectable signals were observed under the same imaging conditions (Figure 6B). In the second HCR, we used S45-SaraFluor488, A161-ATTO550, and S85-Cy5 hairpin DNAs to detect the *Vglut2*, *Penk*, and *Pdyn* mRNAs. *Vglut2*-positive cells were mainly observed in the preoptic area. The *Penk-* or *Pdyn*-expressing cells were densely distributed in and around the striatum and moderately distributed in the preoptic area. Secondary photobleaching also completely quenched the fluorescent HCR labeling (Figure 6B). Additional IHC staining showed specific expression of tyrosine hydroxylase, oxytocin-neurophysin, and nNos in the same section. The immunoreactivity of tyrosine hydroxylase was abundantly observed in the fibers in the striatum, and a small number of cells in the medial preoptic area showed immunoreactivity in their cell bodies. The fibers of oxytocin-neurophysin were sparsely distributed in the preoptic area. nNos-positive cells were observed both in the striatum and in the preoptic area, and the preoptic cells showed a relatively weak signal. These fluorescent stainings were not matched between ISH and IHC (Figure 6C). Thus, by combining two rounds of ISH-HCR combined with IHC, we succeeded in visualizing nine molecules in the same brain section.

**Figure 7:**
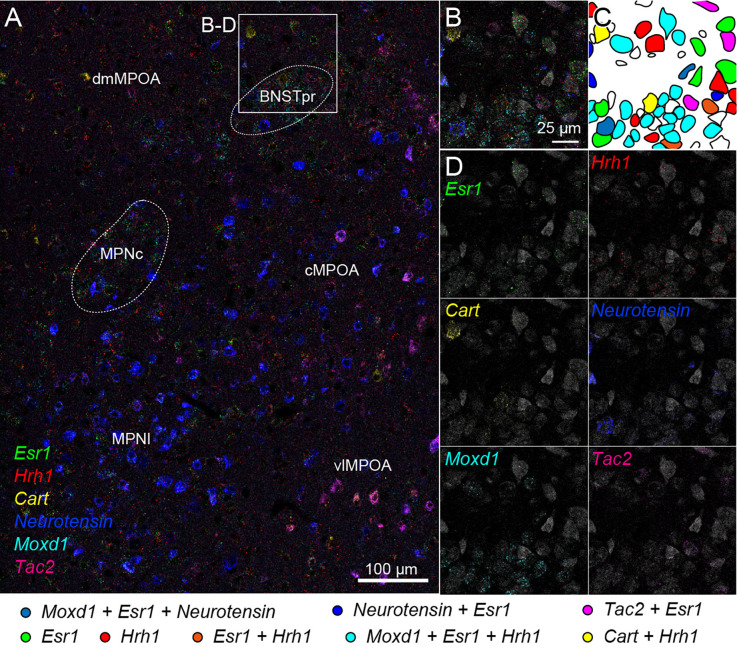
Cell typing using two rounds of ISH combined with Nissl staining. Two-round ISH visualized *Esr1*, *Hrh1*, *Cart*, *neurotensin*, *Moxd1*, and *Tac2* mRNAs. **(A)** Wide-field merged image of the medial preoptic area (MPOA). The rectangle indicates the region shown in **(B–D)**. Scale bar: 100 μm. **(B)** Merged image of six different mRNAs and fluorescent Nissl staining (gray). **(C)** Drawing of each cell position and cell type. Colors indicate different cell types shown at the bottom. **(D)** Separate images of each mRNA and fluorescent Nissl staining. Scale bar: 25 μm. BNSTpr, bed nucleus of the stria terminalis, principal nucleus; cMPOA, central part of MPOA; dmMPOA, dorsomedial part of MPOA; MPNc, medial preoptic nucleus (MPN), core part; MPNl, MPN, lateral part; vlMPOA, ventrolateral MPOA.

We also tried two rounds of ISH using another probe set combined with fluorescent Nissl staining. Region-specific expression of *Esr1*, *Hrh1*, *Cart*, *Neurotensin*, *Moxd1*, and *Tac2* mRNA was observed in the medial preoptic area (MPOA) and divided its subregions (Figure 7A). *Esr1*-positive cells were abundantly found in the central and lateral parts of the medial preoptic nucleus (MPNc and MPNl), the central part of the MPOA (cMPOA), and the bed nucleus of the stria terminalis, the principal component (BNSTpr). *Hrh1* expression was not region specific, but the *Hrh1*-positive cells were not uniformly distributed. *Hrh1*-positive cells were found in the cells that were negative for *Cart, Neurotensin*, or *Tac2* mRNA. *Cart*-positive neurons were found in the dorsomedial and ventrolateral MPOA (dmMPOA and vlMPOA). In the vlMPOA, more than half of the *Cart-*positive neurons were also positive for *Tac2* mRNA. *Neurotensin* mRNA was abundantly found in the MPNl and cMPOA with colocalization of *Esr1* mRNA. *Moxd1*-positive cells were distributed only in the MPNc and BNSTpr and showed highly frequent colocalization of *Esr1* and *Hrh1* mRNAs (Figures 7B–D). *Tac2*-positive cells were found in *Neurotensin*-positive cells in the cMPOA, and *Cart*-positive cells were found in the vlMPOA.

## Discussion

In this study, we developed an LED illuminator with a cooling system that can effectively quench both autofluorescence and fluorescent dyes. High-power LED illumination at a close range drastically and quickly reduced tissue autofluorescence in mouse brains. Quenching autofluorescence led to an increased signal/noise ratio of the ISH-HCR staining, which enabled the detection of very low amounts of mRNAs. Furthermore, fluorescent labeling by ISH-HCR was successfully eliminated without tissue damage by photobleaching using the LED illuminator. These techniques allow highly sensitive multiround ISH combined with IHC for the visualization of multiple targets on the same tissue. Although we showed that this system visualized nine molecules in the same section, the number of molecules can be increased by repeating this labeling-image acquisition-quenching cycle.

The time required for quenching autofluorescence in this system was less than 60 min, the shortest time of any previously reported illuminating device that required 24 h or longer for quenching a wide field as whole slide glass (Schnell et al., 1999; Neumann and Gabel, 2002; Duong and Han, 2013; Sun and Chakrabartty, 2016; Ku et al., 2020). The autofluorescence of thick free-floating sections (40 μm) was also quenched by the same time course (data not shown). Such effectiveness of illumination must require both high-power irradiation and liquid immersion of tissue samples. The color spectrum of LED would be also important to fit the absorption wavelength of lipofuscin. In our setup, slides were illuminated by 60 W/slide, and the distance from LED to tissue was 5 mm. The present condition was optimized based on the quenching effectiveness, and the amount of light received by tissues was distinct among the previous studies listed above. For example, the previous study performed by Duong and Han (2013) illuminated tissue sections at 10 W/slide with LEDs for plant growing, of which the major emission spectrum was biased toward red color. Because of high-power irradiation at 60 W power per specimen, the specimen quickly heats up unless any cooling equipment is used (Figure 1E). Our preliminary experiments showed that the closed container (no opening on its upper side) became too hot after photobleaching and that other cooling systems, such as ice or Peltier cooling, were insufficient or the mass of equipment was too large. Opening the top of the container filled with buffer solution under strong airflow was very simple and effective in suppressing the temperature rise by evaporative cooling. The evaporative cooling system requires the addition of a buffer solution for longer photobleaching (e.g., more than 2 h), but in most cases, sufficient photobleaching should be completed within several hours. An effective cooling system can avoid protein degradation and loss of antigen reactivity caused by heat (Ikeda et al., 2011).

Elimination of autofluorescence of lipofuscin-like granules improved the signal/noise ratio in ISH-HCR staining. Because the size and subcellular location of lipofuscin-like granules were very similar to the fluorescence of mRNA puncta labeled by the ISH techniques detected at a single copy level, it was difficult to distinguish these two. In the control sections of the mouse hippocampus, many fluorescent signals were observed in green, red, and far-red channels, and the distribution of these fluorescent signals was similar among channels, suggesting lipofuscin autofluorescence has a wide emission spectrum (Schnell et al., 1999). These fluorescent signals were eliminated by photobleaching before the HCR staining procedure. Photobleaching increased the high signal/noise ratio except for the blue spectrum and was very effective in visualizing *Drd1* and *Drd2* mRNAs in the hippocampus (Figure 3). *Drd1* and *Drd2* mRNAs were expressed specifically but weakly in the granule cell layer (Tanaka et al., 2011; Sariñana et al., 2014) and polymorphic cell layer of the mouse dentate gyrus (Etter and Krezel, 2014), respectively. These expression patterns were also confirmed by GFP- and Cre-transgenic mice for these genes (Gangarossa et al., 2012; Puighermanal et al., 2017), which is consistent with the present results. In general, the gene expression of G-protein coupled receptors is relatively low, and it is necessary to quench autofluorescence in the brain to accurately determine the expression of such genes. One hour of photobleaching by our LED illuminator should be sufficient for this purpose. The amount of lipofuscin must be varied by multiple biological factors, such as aging, cell types, and species. We observed that the very aged mouse brain accumulated a huge amount of lipofuscin, and in such cases, longer photobleaching was required. Therefore, the duration of irradiation should be decided carefully, especially in the aged primate brain which has very dense lipofuscin granules. We also tested photobleaching by our LED illuminator on the liver, muscle, and lung with fibrosis, which did not contain a huge amount of lipofuscin but showed high autofluorescence. We confirmed the reduction of autofluorescence to detect mRNAs at a single copy level in these tissues (unpublished data). However, the time required to quench autofluorescence depended on the tissue types, extending up to 4 h, which is sufficiently short to prevent degradation of the tissues. Photobleaching seemed to be relatively ineffective in quenching the autofluorescence of erythrocytes.

Our LED illuminator can not only suppress autofluorescence but also quench artificial fluorescent dyes, such as AlexaFluor^®^, Atto^TM^, and Cy^®^ dyes, except for dyes excited by UV. Using this feature, we performed multiround ISH/IHC in a very simple way, consisting of cycles of staining and bleaching. This is the first report of multiround fluorescent ISH using an LED illuminator. Because Hoechst 33342 nuclear staining is relatively tolerated for LED illumination (Figure 4A), additional nuclear staining is usually not necessary. The distribution of gene expression in multiround ISH did not differ from single-round ISH (data not shown) or that in a previous report (Tsuneoka et al., 2017a, b; Moffitt et al., 2018; Tsuneoka and Funato, 2020), suggesting that specific reactions occurred between probes and hairpin DNAs. Although the time required for complete quenching needs to be adjusted depending on the type of fluorophores and abundance of gene expression, two hours of illumination was sufficient for fluorophores in this study. In the mouse striatum, there was a strong expression of *Drd1*, *Drd2*, *Penk*, *Pdyn*, and *Vgat* mRNAs (Lobo et al., 2006; Heiman et al., 2008; Labouesse et al., 2018), and ISH-HCR staining of these mRNAs disappeared after 2 h of photobleaching (Figures 4A, 6B).

To date, several strategies for multiround ISH have been proposed, and quenching fluorophores has not been the major strategy. Although photobleaching by a solid-state laser has been reported, the irradiated region was limited to a very small area (Lubeck et al., 2014; Chen et al., 2015). Another similar approach for multiround ISH is the cleavage of the conjugated fluorophore from DNA. The disulfide bond was used for the conjugation between the fluorophore and DNA readout probes, and the disulfide bond was chemically digested after imaging (Moffitt et al., 2016; Wang et al., 2018). However, such treatment also digests the disulfide bond of peptides in the tissue, which results in tissue damage and makes it unsuitable for IHC. Digesting probes with DNase or stripping hybridized probes by heating have been frequently adopted strategies in other studies (Chen et al., 2016; Shah et al., 2016; Zhu et al., 2018; Eng et al., 2019). This approach for multiround ISH must be useful when the number of available sequences for readout probes is small compared with the required number of ISH rounds. However, additional hybridization steps are required for every round of ISH. We designed 12 sets of short hairpin DNAs, including those in a previous report (Tsuneoka and Funato, 2020; Katayama et al., 2022). Therefore, four rounds of ISH using three colors in each round should be applicable without additional probe hybridization. However, we found that the final round of ISH sometimes showed decreased intensity, regardless of the type of hairpin DNA. These unstable results may be due to nuclease contamination and/or tissue degradation by prolonged incubation of the samples at room temperature. Repeated coverslipping and removing the cover glass sometimes increased the risk of tissue detachment from the slide glass under manual manipulation. Therefore, we recommend three or fewer rounds of ISH/IHC staining practically; otherwise, an automated system or very delicate handling is required to prevent contamination and tissue damage.

## Conclusion

This study showed that the evaporative cooling system-equipped LED illuminator effectively reduced the autofluorescence in nervous tissue within an hour. This photobleaching device drastically improved the signal/noise ratio of fluorescent ISH in a simple way. In addition, the LED illuminator could quench commercially available fluorescent dyes. In combination with the ISH-HCR technique, multiround HCR/IHC, and photobleaching visualize multiple targets in the same tissue samples. The developed protocol will contribute to increasing the quality of histological fluorescent examinations in neuroscience as well as other biological fields.

## Data Availability Statement

The original contributions presented in the study are included in the article/Supplementary material, further inquiries can be directed to the corresponding author/s.

## Ethics Statement

The animal study was reviewed and approved by Institutional Animal Care and Use Committee of Toho University.

## Author Contributions

YT: conceptualization, methodology, and formal analysis. YT, YA, AM, and MY: doing experiments. YT and HF: writing, funding, acquisition, and supervision. All authors contributed to the article and approved the submitted version.

## Funding

This work was supported by the Precise Measurement Technology Promotion Foundation (to YT), JSPS Kakenhi (21K06414 to YT), and Research Grant from Uehara Memorial Foundation (to HF). Part of this research was also performed under financial support by Nepa Gene Corporation.

## Footnotes

^1^ https://blast.ncbi.nlm.nih.gov

## References

[B1] Baharlou H. Canete N. P. Bertram K. M. Sandgren K. J. Cunningham A. L. Harman A. N. . (2021). AFid: a tool for automated identification and exclusion of autofluorescent objects from microscopy images. Bioinformatics 37, 559–567. 10.1093/bioinformatics/btaa780 32931552

[B2] Billinton N. Knight A. W. (2001). Seeing the wood through the trees: a review of techniques for distinguishing green fluorescent protein from endogenous autofluorescence. Anal. Biochem. 291, 175–197. 10.1006/abio.2000.5006 11401292

[B4] Chen K. H. Boettiger A. N. Moffitt J. R. Wang S. Zhuang X. (2015). Spatially resolved, highly multiplexed RNA profiling in single cells. Science 348:aaa6090. 10.1126/science.aaa6090 25858977 PMC4662681

[B3] Chen F. Wassie A. T. Cote A. J. Sinha A. Alon S. Asano S. . (2016). Nanoscale imaging of RNA with expansion microscopy. Nat. Methods 13, 679–684. 10.1038/nmeth.3899 27376770 PMC4965288

[B5] Choi H. M. T. Beck V. A. Pierce N. A. (2014). Next-generation *in situ* hybridization chain reaction: higher gain, lower cost, greater durability. ACS Nano 8, 4284–4294. 10.1021/nn405717p 24712299 PMC4046802

[B6] Choi H. M. T. Chang J. Y. Trinh L. A. Padilla J. E. Fraser S. E. Pierce N. A. (2010). Programmable *in situ* amplification for multiplexed imaging of mRNA expression. Nat. Biotechnol. 28, 1208–1212. 10.1038/nbt.1692 21037591 PMC3058322

[B7] Choi H. M. T. Schwarzkopf M. Fornace M. E. Acharya A. Artavanis G. Stegmaier J. . (2018). Third-generation *in situ* hybridization chain reaction: multiplexed, quantitative, sensitive, versatile, robust. Development 145:dev165753. 10.1242/dev.165753 29945988 PMC6031405

[B8] Clancy B. Cauller L. J. (1998). Reduction of background autofluorescence in brain sections following immersion in sodium borohydride. J. Neurosci. Methods 83, 97–102. 10.1016/s0165-0270(98)00066-1 9765122

[B9] Dirks R. M. Pierce N. A. (2004). Triggered amplification by hybridization chain reaction. Proc. Natl. Acad. Sci. U S A 101, 15275–15278. 10.1073/pnas.0407024101 15492210 PMC524468

[B10] Duong H. Han M. (2013). A multispectral LED array for the reduction of background autofluorescence in brain tissue. J. Neurosci. Methods 220, 46–54. 10.1016/j.jneumeth.2013.08.018 23994358 PMC3856220

[B11] Eng C. H. L. Lawson M. Zhu Q. Dries R. Koulena N. Takei Y. . (2019). Transcriptome-scale super-resolved imaging in tissues by RNA seqFISH+. Nature 568, 235–239. 10.1038/s41586-019-1049-y 30911168 PMC6544023

[B12] Etter G. Krezel W. (2014). Dopamine D2 receptor controls hilar mossy cells excitability. Hippocampus 24, 725–732. 10.1002/hipo.22280 24753432

[B13] Gangarossa G. Longueville S. De Bundel D. Perroy J. Hervé D. Girault J. A. . (2012). Characterization of dopamine D1 and D2 receptor-expressing neurons in the mouse hippocampus. Hippocampus 22, 2199–2207. 10.1002/hipo.22044 22777829

[B14] Heiman M. Schaefer A. Gong S. Peterson J. D. Day M. Ramsey K. E. . (2008). A translational profiling approach for the molecular characterization of CNS cell types. Cell 135, 738–748. 10.1016/j.cell.2008.10.028 19013281 PMC2696821

[B15] Ikeda K. Suzuki T. Tate G. Mitsuya T. (2011). Multiple immunoenzyme labeling using heat treatment combined with the polymer method: an analysis of the appropriate inactivation conditions of primary antibodies. Acta Histochem. 113, 117–124. 10.1016/j.acthis.2009.08.007 19775731

[B16] Ivy G. O. Schottler F. Wenzel J. Baudry M. Lynch G. (1984). Inhibitors of lysosomal enzymes: accumulation of lipofuscin-like dense bodies in the brain. Science 226, 985–987. 10.1126/science.6505679 6505679

[B17] Katayama Y. Saito A. Ogoshi M. Tsuneoka Y. Mukuda T. Azuma M. . (2022). Gene duplication of C-type natriuretic peptide-4 (CNP4) in teleost lineage elicits subfunctionalization of ancestral CNP. Cell Tissue Res. 388, 225–238. 10.1007/s00441-022-03596-y 35171324

[B18] Ku T. Guan W. Evans N. B. Sohn C. H. Albanese A. Kim J. G. . (2020). Elasticizing tissues for reversible shape transformation and accelerated molecular labeling. Nat. Methods 17, 609–613. 10.1038/s41592-020-0823-y 32424271 PMC8056749

[B19] Labouesse M. A. Sartori A. M. Weinmann O. Simpson E. H. Kellendonk C. Weber-Stadlbauer U. (2018). Striatal dopamine 2 receptor upregulation during development predisposes to diet-induced obesity by reducing energy output in mice. Proc. Natl. Acad. Sci. U S A 115, 10493–10498. 10.1073/pnas.1800171115 30254156 PMC6187139

[B20] Larsson C. Grundberg I. Söderberg O. Nilsson M. (2010). *In situ* detection and genotyping of individual mRNA molecules. Nat. Methods 7, 395–397. 10.1038/nmeth.1448 20383134

[B21] Lobo M. K. Karsten S. L. Gray M. Geschwind D. H. Yang X. W. (2006). FACS-array profiling of striatal projection neuron subtypes in juvenile and adult mouse brains. Nat. Neurosci. 9, 443–452. 10.1038/nn1654 16491081

[B22] Lubeck E. Coskun A. F. Zhiyentayev T. Ahmad M. Cai L. (2014). Single-cell *in situ* RNA profiling by sequential hybridization. Nat. Methods 11, 360–361. 10.1038/nmeth.2892 24681720 PMC4085791

[B23] Maejima T. Tsuno Y. Miyazaki S. Tsuneoka Y. Hasegawa E. Islam M. T. . (2021). GABA from vasopressin neurons regulates the time at which suprachiasmatic nucleus molecular clocks enable circadian behavior. Proc. Natl. Acad. Sci. U S A 118:e2010168118. 10.1073/pnas.2010168118 33526663 PMC8017960

[B24] Maynard K. R. Tippani M. Takahashi Y. Phan B. D. N. Hyde T. M. Jaffe A. E. . (2021). *dotdotdot*: an automated approach to quantify multiplex single molecule fluorescent in situ hybridization (smFISH) images in complex tissues. Nucleic Acids Res. 48:e66. 10.1093/nar/gkaa312 32383753 PMC7293004

[B25] Moffitt J. R. Bambah-Mukku D. Eichhorn S. W. Vaughn E. Shekhar K. Perez J. D. . (2018). Molecular, spatial and functional single-cell profiling of the hypothalamic preoptic region. Science 362:eaau5324. 10.1126/science.aau5324 30385464 PMC6482113

[B26] Moffitt J. R. Hao J. Bambah-Mukku D. Lu T. Dulac C. Zhuang X. (2016). High-performance multiplexed fluorescence in situ hybridization in culture and tissue with matrix imprinting and clearing. Proc. Natl. Acad. Sci. U S A 113, 14456–14461. 10.1073/pnas.1617699113 27911841 PMC5167177

[B27] Neumann M. Gabel D. (2002). Simple method for reduction of autofluorescence in fluorescence microscopy. J. Histochem. Cytochem. 50, 437–439. 10.1177/002215540205000315 11850446

[B28] O’Connell M. J. Baum H. Peters T. J. (1986). Haemosiderin-like properties of free-radical-modified ferritin. Biochem. J. 240, 297–300. 10.1042/bj2400297 3827850 PMC1147411

[B29] Player A. N. Shen L. P. Kenny D. Antao V. P. Kolberg J. A. (2001). Single-copy gene detection using branched DNA (bDNA) *in situ* hybridization. J. Histochem. Cytochem. 49, 603–612. 10.1177/002215540104900507 11304798

[B30] Puighermanal E. Cutando L. Boubaker-Vitre J. Honoré E. Longueville S. Hervé D. . (2017). Anatomical and molecular characterization of dopamine D1 receptor-expressing neurons of the mouse CA1 dorsal hippocampus. Brain Struct. Funct. 222, 1897–1911. 10.1007/s00429-016-1314-x 27678395 PMC5406422

[B31] Pyon W. S. Gray D. T. Barnes C. A. (2019). An alternative to dye-based approaches to remove background autofluorescence from primate brain tissue. Front. Neuroanat. 13:73. 10.3389/fnana.2019.00073 31379520 PMC6657503

[B32] Rocco B. R. Oh H. Shukla R. Mechawar N. Sibille E. (2017). Fluorescence-based cell-specific detection for laser-capture microdissection in human brain. Sci. Rep. 7:14213. 10.1038/s41598-017-14484-9 29079825 PMC5660154

[B33] Sariñana J. Kitamura T. Künzler P. Sultzman L. Tonegawa S. (2014). Differential roles of the dopamine 1-class receptors, D1R and D5R, in hippocampal dependent memory. Proc. Natl. Acad. Sci. U S A 111, 8245–8250. 10.1073/pnas.1407395111 24843151 PMC4050601

[B34] Schnell S. A. Staines W. A. Wessendorf M. W. (1999). Reduction of lipofuscin-like autofluorescence in fluorescently labeled tissue. J. Histochem. Cytochem. 47, 719–730. 10.1177/002215549904700601 10330448

[B35] Shah S. Lubeck E. Zhou W. Cai L. (2016). *In situ* transcription profiling of single cells reveals spatial organization of cells in the mouse hippocampus. Neuron 92, 342–357. 10.1016/j.neuron.2016.10.001 27764670 PMC5087994

[B36] Sun Y. Chakrabartty A. (2016). Cost-effective elimination of lipofuscin fluorescence from formalin-fixed brain tissue by white phosphor light emitting diode array. Biochem. Cell Biol. 94, 545–550. 10.1139/bcb-2016-0125 27824490

[B37] Tanaka T. Kai N. Kobayashi K. Takano Y. Hironaka N. (2011). Up-regulation of dopamine D1 receptor in the hippocampus after establishment of conditioned place preference by cocaine. Neuropharmacology 61, 842–848. 10.1016/j.neuropharm.2011.05.032 21669213

[B38] Tsuneoka Y. Funato H. (2020). Modified *in situ* hybridization chain reaction using short hairpin DNAs. Front. Mol. Neurosci. 13:75. 10.3389/fnmol.2020.00075 32477063 PMC7235299

[B39] Tsuneoka Y. Tsukahara S. Yoshida S. Takase K. Oda S. Kuroda M. . (2017a). Moxd1 is a marker for sexual dimorphism in the medial preoptic area, bed nucleus of the stria terminalis and medial amygdala. Front. Neuroanat. 11:26. 10.3389/fnana.2017.00026 28396628 PMC5366752

[B40] Tsuneoka Y. Yoshida S. Takase K. Oda S. Kuroda M. Funato H. (2017b). Neurotransmitters and neuropeptides in gonadal steroid receptor-expressing cells in medial preoptic area subregions of the male mouse. Sci. Rep. 7:9809. 10.1038/s41598-017-10213-4 28852050 PMC5575033

[B41] Wang F. Flanagan J. Su N. Wang L. C. Bui S. Nielson A. . (2012). RNAscope: a novel *in situ* RNA analysis platform for formalin-fixed, paraffin-embedded tissues. J. Mol. Diagn. 14, 22–29. 10.1016/j.jmoldx.2011.08.002 22166544 PMC3338343

[B42] Wang G. Moffitt J. R. Zhuang X. (2018). Multiplexed imaging of high-density libraries of RNAs with MERFISH and expansion microscopy. Sci. Rep. 8:4847. 10.1038/s41598-018-22297-7 29555914 PMC5859009

[B43] Zhu Q. Shah S. Dries R. Cai L. Yuan G. C. (2018). Identification of spatially associated subpopulations by combining scRNAseq and sequential fluorescence *in situ* hybridization data. Nat. Biotechnol. 36, 1183–1190. 10.1038/nbt.4260. [Online ahead of print]. 30371680 PMC6488461

